# Association between nutritional factors and myopia in adolescents: a systematic review and meta-analysis

**DOI:** 10.3389/fpubh.2025.1670103

**Published:** 2025-10-01

**Authors:** Zhaoxia Xu

**Affiliations:** School of Stomatology and Optometry, Hubei University of Science and Technology, Xianning, Hubei, China

**Keywords:** myopia, adolescents, nutritional factors, dietary intake, systematic review

## Abstract

**Background:**

Myopia is a highly prevalent eye disorder among adolescents, and an increasing body of research indicates that nutritional factors may have a significant impact on its development. However, the nature and extent of these relationships remain unclear. This systematic review and meta-analysis aimed to comprehensively evaluate the associations between various nutritional factors, including carbohydrates, proteins, cholesterol, and sodium, and myopia in adolescents.

**Methods:**

Multiple databases, such as PubMed, Web of Science, Scopus, and Embase, were systematically searched up to February 15, 2025. The inclusion criteria encompassed observational studies published in English, involving adolescents (aged 6–18 years), and reporting data on the intake of the selected nutritional factors and myopia status. Two reviewers independently screened studies, extracted data, and assessed study quality using the Newcastle-Ottawa Scale. Standardized mean differences (SMDs) with 95% confidence intervals (CIs) were calculated for continuous outcomes. Random-effects models were applied to account for potential heterogeneity.

**Results:**

A total of 7 articles (8 studies) involving 45,993 adolescents were included. Pooled analysis revealed significant associations between nutritional factors and myopia risk. Higher carbohydrate intake was positively linked to myopia (SMD = 0.36, 95% CI: 0.22–0.50, I^2^ = 94.8%, *p* < 0.001), while protein intake showed a protective effect (SMD = −0.25, 95% CI: −0.27 to −0.23, I^2^ = 44.0%, *p* < 0.001). Cholesterol intake was associated with increased myopia risk (SMD = 0.20, 95% CI: 0.10–0.31, I^2^ = 91.7%, *p* < 0.001), and sodium intake demonstrated a strong positive association (SMD = 1.07, 95% CI: 0.93–1.22, I^2^ = 96.0%, *p* < 0.001). Sensitivity analyses confirmed the robustness of results, and no publication bias was detected.

**Conclusion:**

This study suggests potential associations between nutritional factors and myopia in adolescents. Carbohydrates, cholesterol, and sodium were positively associated with myopia, whereas proteins showed a possible protective effect. However, given the small number of available studies, the predominance of cross-sectional designs, and substantial heterogeneity, these findings should be considered preliminary. Future well-designed, longitudinal or interventional studies are required to confirm these associations before any firm dietary recommendations can be made for myopia prevention.

## Introduction

Myopia, one of the most prevalent refractive errors, is characterized by impaired distance vision and has reached epidemic proportions worldwide, particularly among adolescents (1). Recent estimates indicate that approximately 30–50% of children and adolescents in East Asia are affected, with projections suggesting that by 2050, nearly half of the global population may be myopic (2–4). Beyond its immediate impact on visual acuity, myopia is associated with an increased risk of severe ocular complications, including retinal detachment, glaucoma, and cataracts, underscoring the urgent need for effective prevention strategies during childhood and adolescence (5, 6).

Well-established determinants of myopia progression include genetic predisposition, reduced outdoor activity, and prolonged near work or screen time, which together explain a large proportion of the current epidemic, particularly in East Asia (2). Nevertheless, these factors alone do not fully account for the rapid rise in prevalence, suggesting that additional modifiable influences may contribute.

Within this broader etiological framework, dietary factors have recently attracted increasing attention. Nutrition may interact with genetic and behavioral determinants to influence eye growth and refractive development through pathways involving insulin-like growth factor 1 (IGF-1) signaling, oxidative stress, and inflammation (4, 6). Situating nutrition alongside established factors such as outdoor exposure and digital device use provides a more comprehensive perspective on the multifactorial origins of myopia and underscores the potential relevance of investigating dietary contributions.

Within this broader etiological framework, nutrition has recently gained attention as a potentially modifiable factor. Emerging evidence suggests that dietary components, including macronutrients (carbohydrates, proteins, fats) and micronutrients (vitamins, minerals), may influence eye growth and refractive development via multiple mechanisms, such as modulation of IGF-1 signaling, oxidative stress, and inflammatory pathways (7–9). Nevertheless, studies investigating the association between specific nutrients and adolescent myopia have yielded conflicting results. For instance, some cross-sectional studies suggest that a high carbohydrate intake may elevate myopia risk by inducing hyperglycemic responses and accelerating axial elongation (10, 11), whereas others report no significant associations (12). Similarly, protein-rich diets have been postulated to confer protective effects by supporting collagen synthesis and maintaining scleral integrity (13, 14), yet the evidence remains inconsistent.

To address these knowledge gaps, this systematic review and meta-analysis aim to comprehensively synthesize current evidence on the associations between key nutritional factors—including carbohydrates, proteins, cholesterol, and sodium—and myopia in adolescents. By integrating data from observational studies, we seek to quantify the magnitude of these associations, identify potential sources of heterogeneity, and establish a more robust foundation for future research and public health interventions.

## Materials and methods

### Data sources and search strategy

**Figure 1:**
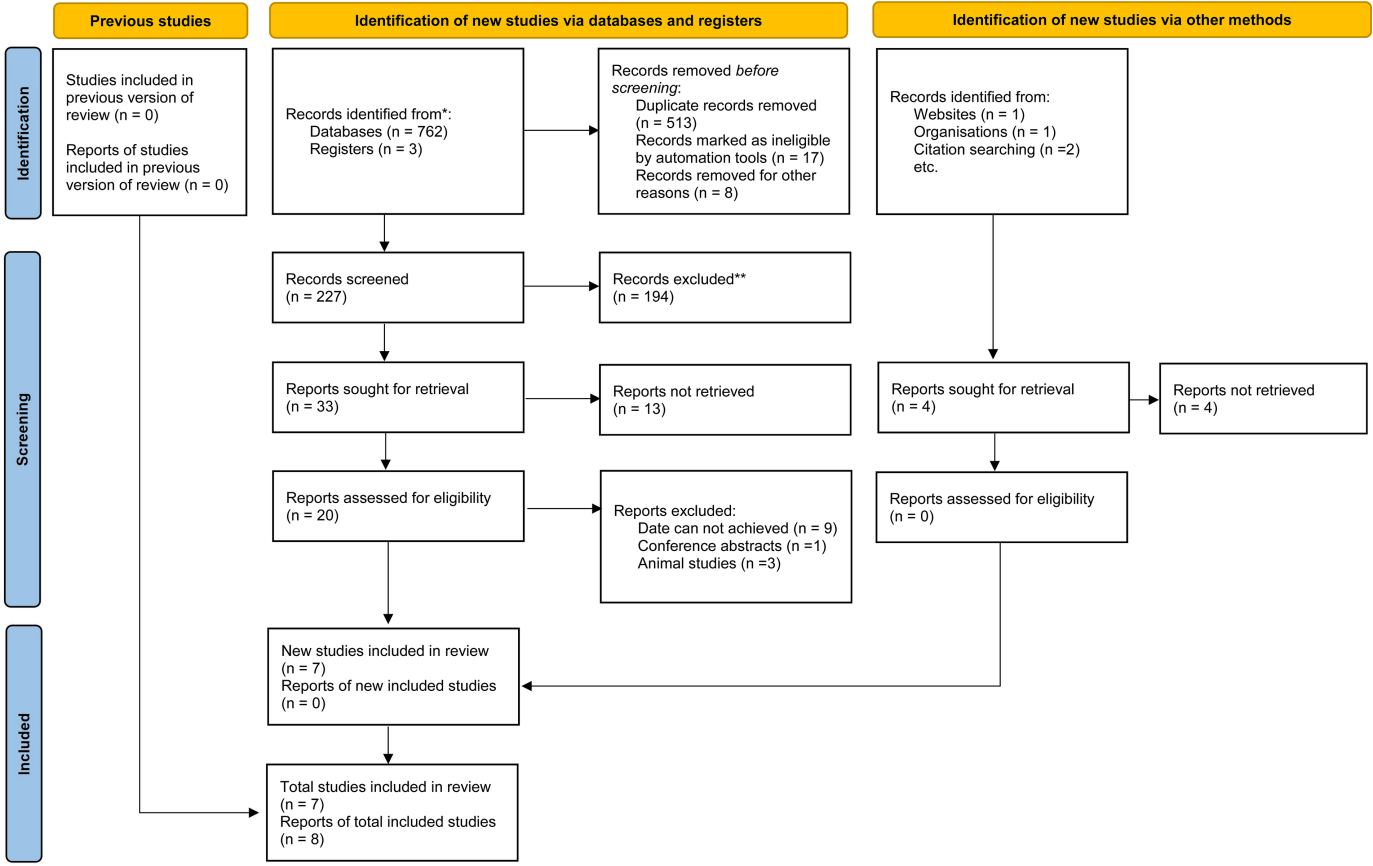
Flowchart of study selection process.

A systematic search was conducted across four databases: PubMed, Web of Science, Scopus, and Embase, up to February 15, 2025. The search strategy combined keywords related to nutritional factors (carbohydrates, proteins, cholesterol, sodium, dietary fats, vitamins, minerals), myopia (myopia, nearsightedness, refractive error), and adolescents (adolescents, children, juveniles, young adults). Detailed search terms for each database are listed in Table 1 and Figure 1.

**Table 1:** Search strategy.

Database	Search strategy
Pubmed	((“Carbohydrates”[Mesh] OR carbohydrates[Title/Abstract] OR “Proteins”[Mesh] OR proteins[Title/Abstract] OR “Cholesterol”[Mesh] OR cholesterol[Title/Abstract] OR “Sodium”[Mesh] OR sodium[Title/Abstract] OR “Dietary Fats”[Mesh] OR fats[Title/Abstract] OR “Vitamins”[Mesh] OR vitamins[Title/Abstract] OR “Minerals”[Mesh] OR minerals[Title/Abstract]) AND (“Myopia”[Mesh] OR myopia[Title/Abstract] OR “Nearsightedness”[Mesh] OR nearsightedness[Title/Abstract] OR “Refractive Errors”[Mesh] OR “refractive error*”[Title/Abstract])) AND (“Adolescent”[Mesh] OR adolescents[Title/Abstract] OR “Child”[Mesh] OR children[Title/Abstract] OR “Juvenile”[Mesh] OR juveniles[Title/Abstract] OR “Young Adult”[Mesh] OR “young adult*”[Title/Abstract])
Embase	(‘carbohydrate’:ab,ti,kw OR ‘protein’:ab,ti,kw OR ‘cholesterol’:ab,ti,kw OR ‘sodium’:ab,ti,kw OR ‘dietary fat’:ab,ti,kw OR ‘vitamin’:ab,ti,kw OR ‘mineral’:ab,ti,kw) AND (‘myopia’:ab,ti,kw OR ‘nearsightedness’:ab,ti,kw OR ‘refractive error’:ab,ti,kw) AND (‘adolescent’:ab,ti,kw OR ‘child’:ab,ti,kw OR ‘juvenile’:ab,ti,kw OR ‘young adult’:ab,ti,kw)
Scopus	TITLE-ABS-KEY((“carbohydrate*” OR “protein*” OR “cholesterol*” OR “sodium*” OR “dietary fat*” OR “vitamin*” OR “mineral*”) AND (“myopia” OR “nearsightedness” OR “refractive error*”)) AND TITLE-ABS-KEY((“adolescent*” OR “child*” OR “juvenile*” OR “young adult*”))
Web of Science	TS = ((carbohydrate* OR protein* OR cholesterol* OR sodium* OR dietary fat* OR vitamin* OR mineral*) AND (myopia OR nearsightedness OR refractive error*)) AND TS = (adolescent* OR child* OR juvenile* OR young adult*) AND DT = (Article OR Review OR Meta-Analysis)

### Inclusion and exclusion criteria

Inclusion criteria: (1) Observational studies (cross-sectional, cohort) evaluating the association between dietary intake (carbohydrates, proteins, cholesterol, sodium) and myopia in adolescents aged 6–18 years. (2) Studies reporting quantitative data (e.g., mean intake, effect estimates) for at least one nutritional factor. (3) Myopia defined as spherical equivalent (SE) ≤ −0.50 D or cycloplegic refraction ≤ − 0.50 D. Exclusion criteria: (1) Interventional studies, case reports, reviews, or studies without original data. (2) Studies focusing on other refractive errors (e.g., hyperopia) or non-adolescent populations.

Although our search strategy was intentionally broad (including fats, vitamins, and minerals), only carbohydrates, proteins, cholesterol, and sodium had sufficient and comparable data across multiple studies to allow quantitative synthesis. Other nutrients were excluded from the pooled analysis due to the paucity of eligible studies, but they remain important topics for future research.

### Study selection and data extraction

Two authors independently screened titles/abstracts and full texts for eligibility. Discrepancies were resolved through consensus. Data extracted included: (1) Study characteristics: Country, design, sample size, mean age, and NOS score. (2) Dietary assessment methods: Food frequency questionnaires (FFQ), 24-h dietary recall, or semi-quantitative diaries. (3) Myopia definition: SE thresholds and cycloplegic requirements. (4) Key findings: Mean nutrient intake (g/day or mg/day) in myopic vs. non-myopic groups. Full details of included studies are summarized in Tables 2–5.

**Table 2:** Characteristics of studies evaluating carbohydrates intake and myopia in adolescents.

First author, year	Country	Study design	Sample size (n)	Mean age	Comparative intervention approaches	Dietary assessment	Definition of myopia	Key findings	NOS score
Berticat et al. (2020) (16)	France	Cross-sectional	88	9.5 years	Girls: Amount of carbohydrates intake (g/day) in myopic and Non-myopic groups: 1. myopic (*n* = 49): 181.94 ± 13.56 g/day; 2. Non-myopic (*n* = 39): 175.04 ± 10.06 g/day	Food frequency questionnaire (refined carbohydrates intake)	Refraction <0 D in ≥1 eye	Girls: Refined carbohydrates consumption increased myopia risk	8
Berticat et al. (2020) (16)	France	Cross-sectional	92	9.5 years	Boys: Amount of carbohydrates intake (g/day) in myopic and Non-myopic groups: 1. myopic (*n* = 37): 178.03 ± 12.04 g/day; 2. Non-myopic (*n* = 55): 164.78 ± 10.98 g/day	Food frequency questionnaire (refined carbohydrates intake)	Refraction <0 D in ≥1 eye	Boys: Refined carbohydrates consumption increased myopia risk	8
Chua et al. (2018) (20)	Singapore	Cohort	317	36.5 months	Comparison of nutrient intake (g/day) between the first and third tertiles: 1. myopic (*n* = 185): 44.16 ± 13.25 g/day; 2. Non-myopic (*n* = 132): 42.84 ± 11.21 g/day	A three-day food diary	SE ≤ − 0.50 D	There was no significant association between the intake of carbohydrates and myopia	7
Kim et al. (2024) (9)	Korea	Cross-sectional	18,077	15.05 years	Daily carbohydrate intake (g/day) comparison between myopic and non-myopic groups: 1. Myopic (*n* = 15,843): 342.18 ± 20.45 g/day 2. Non-myopic (*n* = 2,234): 335.91 ± 18.21 g/day	24-h personalized dietary recall method	SE ≤ −0.50 D	Higher carbohydrate intake associated with increased myopia risk	8
Kim et al. 2024 (18)	Korea	Cross-sectional	24,345	9.00 years	Daily nutrient intake (g/day) comparison between myopic and non-myopic children: 1. Myopic (*n* = 14,944): - carbohydrates: 298.04 ± 5.94 g/day 2. Non-myopic (*n* = 9,401): - carbohydrates: 291.88 ± 4.22 g/day	24-h personalized dietary recall method	SE ≤ −0.50 D	High carbohydrates linked to higher myopia risk.	8
Li et al. (2022) (21)	Singapore	Cohort	467	9.00 years	Daily nutrient intake comparison between myopic and non-myopic children: 1. Myopic (*n* = 258): - carbohydrates: 253.9 ± 6.15 g/day 2. Non-myopic (*n* = 209): - carbohydrates: 242.6 ± 7.83 g/day	Semi-quantitative	SE ≤ −0.50 D	Findings showed no significant association between carbohydrates intake and myopia	7
Lim et al. (2010) (22)	Singapore	Cross-sectional	851	12.81 years	Myopic (*n* = 431): - carbohydrates: 417.8 ± 7.81 g/day 2. Non-myopic (*n* = 420): - carbohydrates: 403.2 ± 8.84 g/day	Semi-quantitative	SE ≤ −0.50 D	Higher intake of carbohydrates was associated with myopia	6
Sun et al. (2024) (19)	China	Cross-sectional	1756	8.76 ± 2.06 years	Myopic (*n* = 1,276): - carbohydrates: 341.6 ± 4.95 g/day 2. Non-myopic (*n* = 480): - carbohydrates: 330.9 ± 7.01 g/day	Questionnaire-based dietary habits	Cycloplegic SER ≤ −0.50 D	High-carbohydrates diet was key risk factor.	8

“-” indicates that no relevant information or data was obtained.

**Table 3:** Characteristics of studies evaluating protein intake and myopia in adolescents.

First author, Year	Country	Study design	Sample size (n)	Mean age	Comparative intervention approaches	Dietary assessment	Definition of myopia	Key findings	NOS score
Chua et al. (2018) (20)	Singapore	Cohort	317	36.5 months	Comparison of protein intake between the first and third tertiles: 1. myopic (*n* = 185): 38.81 ± 0.87 g/day; 2. Non-myopic (*n* = 132): 39.43 ± 1.20 g/day	A three-day food diary	SE ≤ − 0.50 D	There was no significant association between the intake of protein and myopia	7
Kim et al. (2024) (9)	Korea	Cross-sectional	18,077	15.05 years	Daily protein intake (g/day) comparison between myopic and non-myopic groups: 1. Myopic (*n* = 15,843): 80.85 ± 0.53 g/day 2. Non-myopic (*n* = 2,234): 84.74 ± 0.20 g/day	24-h dietary recall	SE ≤ −0.50 D in right eye	Higher protein intake associated with decreased myopia risk	8
Kim et al. (2024) (18)	Korea	Cross-sectional	24,345	9.00 years	Daily protein intake (g/day) comparison between myopic and non-myopic groups: 1. Myopic (*n* = 14,944): 65.99 ± 0.40 g/day 2. Non-myopic (*n* = 9,401): 66.67 ± 0.13 g/day	24-h dietary recall	SE ≤ −0.50 D in right eye	Higher protein intake associated with decreased myopia risk	8
Li et al. (2022) (21)	Singapore	Cohort	467	9.00 years	Daily protein intake comparison between myopic and non-myopic children: 1. Myopic (*n* = 258): - protein: 52.9 ± 3.63 g/day 2. Non-myopic (*n* = 209): - protein: 54.8 ± 4.17 g/day	Semi-quantitative	SE ≤ −0.50 D	Higher protein intake associated with decreased myopia risk	7
Lim et al. (2010) (22)	Singapore	Cross-sectional	851	12.81 years	Myopic (n = 431): - protein: 40.8 ± 2.84 g/day 2. Non-myopic (*n* = 420): - protein: 42.2 ± 3.46 g/day	Semi-quantitative	SE ≤ −0.50 D	Higher protein intake associated with decreased myopia risk	6

Note: “-” indicates that no relevant information or data was obtained.

**Table 4:** Characteristics of studies evaluating cholesterol intake and myopia in adolescents.

First author, Year	Country	Study design	Sample size (n)	Mean age	Comparative intervention approaches	Dietary assessment	Definition of myopia	Key findings	NOS score
Kim et al. (2024) (9)	Korea	Cross-sectional	18,077	15.05 years	Daily cholesterol intake (mg/day) comparison between myopic and non-myopic groups: 1. Myopic (*n* = 15,843): 349.68 ± 1.58 mg/day 2. Non-myopic (*n* = 2,234): 330.92 ± 4.24 mg/day	24-h dietary recall	SE ≤ −0.50 D in right eye	Higher cholesterol intake associated with increased myopia risk	8
Kim et al. (2024) (18)	Korea	Cross-sectional	24,345	9.00 years	Daily cholesterol intake (mg/day) comparison between myopic and non-myopic groups: 1. Myopic (*n* = 14,944): 300.32 ± 1.66 mg/day 2. Non-myopic (*n* = 9,401): 278.19 ± 2.31 mg/day	24-h dietary recall	SE ≤ −0.50 D in right eye	Higher cholesterol intake associated with increased myopia risk	8
Lim et al. (2010) (22)	Singapore	Cross-sectional	851	12.81 years	Myopic (*n* = 431): - cholesterol: 452.6 ± 3.42 mg/day 2. Non-myopic (*n* = 420): - cholesterol: 436.4 ± 3.07 mg/day	Semi-quantitative	SE ≤ −0.50 D	Higher intake of cholesterol was associated with myopia	6
Sun et al. (2024) (19)	China	Cross-sectional	1,756	8.76 ± 2.06 years	Daily cholesterol intake (mg/day) comparison between myopic and non-myopic groups: 1. Myopic (*n* = 1,276): 295.15 ± 1.44 mg/day 2. Non-myopic (*n* = 480): 273.06 ± 1.78 mg/day	Questionnaire-based dietary habits	Cycloplegic SER ≤ −0.50 D	High-cholesterol diet and parental myopia were key risk factors across age groups.	8

“-” indicates that no relevant information or data was obtained.

**Table 5:** Characteristics of studies evaluating sodium intake and myopia in adolescents.

First author, Year	Country	Study design	Sample size (n)	Mean age	Comparative intervention approaches	Dietary assessment	Definition of myopia	Key findings	NOS score
Kim et al. (2024) (9)	Korea	Cross-sectional	18,077	15.05 years	Daily sodium intake (mg/day) comparison between myopic and non-myopic groups: 1. Myopic (*n* = 15,843): 3739.94 ± 9.93 mg/day 2. Non-myopic (*n* = 2,234): 3536.66 ± 26.65 mg/day	24-h dietary recall	SE ≤ −0.50 D in right eye	Higher sodium intake associated with increased myopia risk	8
Kim et al. (2024) (18)	Korea	Cross-sectional	24,345	9.00 years	Daily sodium intake (mg/day) comparison between myopic and non-myopic groups: 1. Myopic (*n* = 14,944): 2912.65 ± 8.82 mg/day 2. Non-myopic (*n* = 9,401): 2644.88 ± 12.24 mg/day	24-h dietary recall	SE ≤ −0.50 D in right eye	Higher sodium intake associated with increased myopia risk	8
Sun et al. (2024) (19)	China	Cross-sectional	1,756	8.76 ± 2.06 years	Daily sodium intake (mg/day) comparison between myopic and non-myopic groups: 1. Myopic (*n* = 1,276): 2743.84 ± 9.25 mg/day 2. Non-myopic (*n* = 480): 2528.09 ± 10.16 mg/day	Questionnaire-based dietary habits	Cycloplegic SER ≤ −0.50 D	High-sodium diet was key risk factor of myopia	8

“-” indicates that no relevant information or data was obtained.

### Quality assessment

The quality of each included study was assessed using the Newcastle-Ottawa Scale (NOS) (15), which evaluates three key methodological domains in observational studies: selection of the study population, comparability of study groups, and outcome assessment. For cohort studies, the assessment criteria included the representativeness of the exposed cohort, the selection of an appropriate non-exposed cohort, and the adequacy of follow-up. In case–control studies, key factors such as the definition of cases and controls, the selection of control groups, and the assessment of exposure were considered. For cross-sectional studies, the evaluation focused on sampling methods, population definition, and the assessment of both exposure and outcome measures. Each study was assigned a score ranging from 0 to 9, with higher scores indicating better methodological rigor. Studies scoring ≥7 stars were classified as high-quality.

### Statistical analysis

Standardized mean differences (SMDs) with 95% confidence intervals (CIs) were calculated for continuous outcomes (nutrient intake) using the inverse variance method, ensuring comparability across studies with different measurement units (e.g., grams/day vs. milligrams/day). Heterogeneity among studies was assessed using the Cochrane Q statistic and the I^2^ statistic, where a significant Q test (*p* < 0.05) indicated the presence of heterogeneity, and an I^2^ value greater than 50% suggested substantial heterogeneity. Given the substantial heterogeneity observed in preliminary analyses, we conducted predefined subgroup analyses according to study design (cross-sectional *vs.* cohort), dietary assessment method (24-h recall *vs.* FFQ *vs.* semi-quantitative diary), and study region (East Asia *vs.* Western countries). Meta-regression analyses were also performed where possible to further explore potential sources of heterogeneity. Sensitivity analyses were performed by sequentially excluding each study from the meta-analysis to evaluate the robustness of the pooled effect estimates and identify studies with disproportionate influence. Publication bias was assessed through visual inspection of funnel plot asymmetry and formally tested using Begg’s regression test, with *p* < 0.05 indicating potential bias. All statistical analyses were performed using Stata 17.0 (StataCorp, College Station, TX, USA) and Review Manager 5.4 (Cochrane Collaboration, Copenhagen, Denmark).

## Results

### Characteristics of included studies

A total of 7 articles (comprising 8 studies) (16–22) were included in the meta-analysis, involving 45,993 adolescents aged 6–18 years. The studies evaluated associations between carbohydrates (16–22) (8 studies, 45,993 participants), proteins (17, 18, 20–22) (5 studies, 44,057 participants), cholesterol (17–19, 22) (4 studies, 45,029 participants), and sodium (17–19) (3 studies, 44,178 participants) intake and myopia risk. Study designs included cross-sectional (6 studies) and cohort (2 studies) approaches. Dietary assessments varied, with 24-h dietary recalls (4 studies), food frequency questionnaires (3 studies), and semi-quantitative diaries (1 study) used. Study quality scores ranged from 6 to 8 stars on the NOS (Tables 2–5).

### Carbohydrate intake and myopia risk

**Figure 2:**
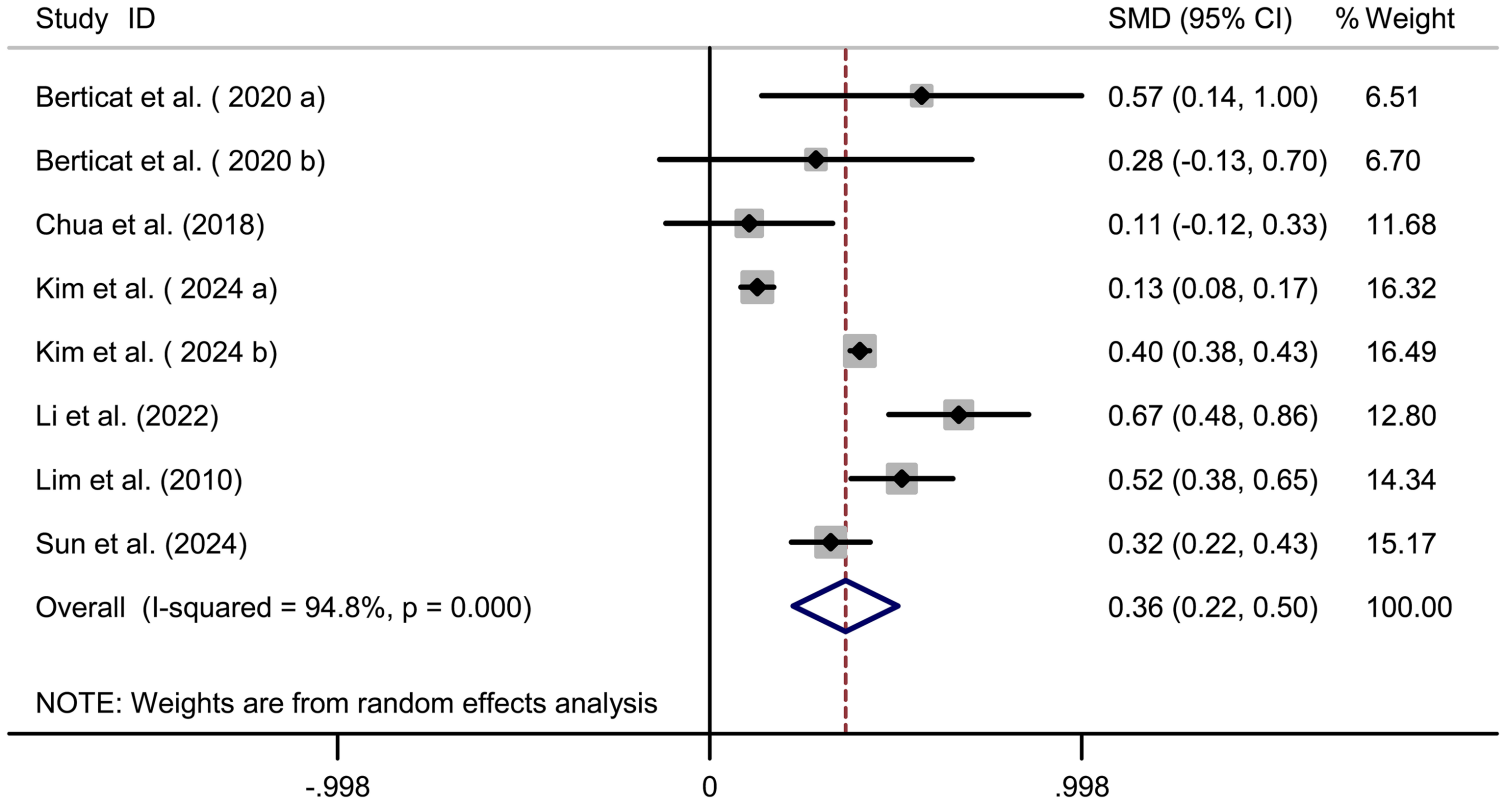
Forest plot of carbohydrate intake and myopia risk.

**Figure 3:**
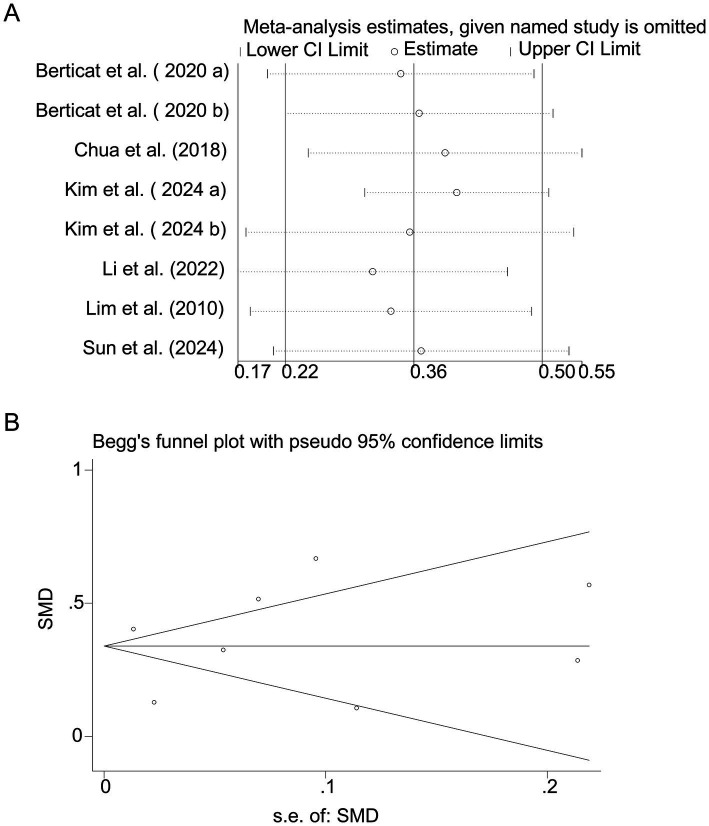
Sensitivity analysis and funnel plot for carbohydrate intake. **(A)** Sensitivity analysis for carbohydrate intake. **(B)** Funnel plot for carbohydrate intake publication bias.

Pooled analysis of 7 articles (comprising 8 studies) (16–22) revealed a significant positive association between carbohydrate intake and myopia risk (SMD = 0.36, 95% CI: 0.22–0.50, I^2^ = 94.8%, *p* < 0.001; Figure 2). Subgroup analyses indicated that the very high heterogeneity (I^2^ = 94.8%) was partly attributable to study region and dietary assessment method. East Asian studies (Korea, China, Singapore) demonstrated a stronger positive association between carbohydrate intake and myopia (SMD = 0.38, 95% CI: 0.24–0.52; I^2^ = 88.2%), whereas the French cohorts showed weaker associations (SMD = 0.12, 95% CI: −0.05–0.29; I^2^ = 42.1%). Heterogeneity was also markedly reduced in studies using semi-quantitative dietary diaries or 24-h recalls (I^2^ = 70.4%) compared with those relying solely on food frequency questionnaires (I^2^ = 95.3%). Sensitivity analysis excluding individual studies did not alter the overall effect estimate (Figure 3A), indicating robustness. Funnel plot symmetry and Begg’s test (*p* = 0.12) suggested no publication bias (Figure 3B).

### Protein intake and myopia risk

**Figure 4:**
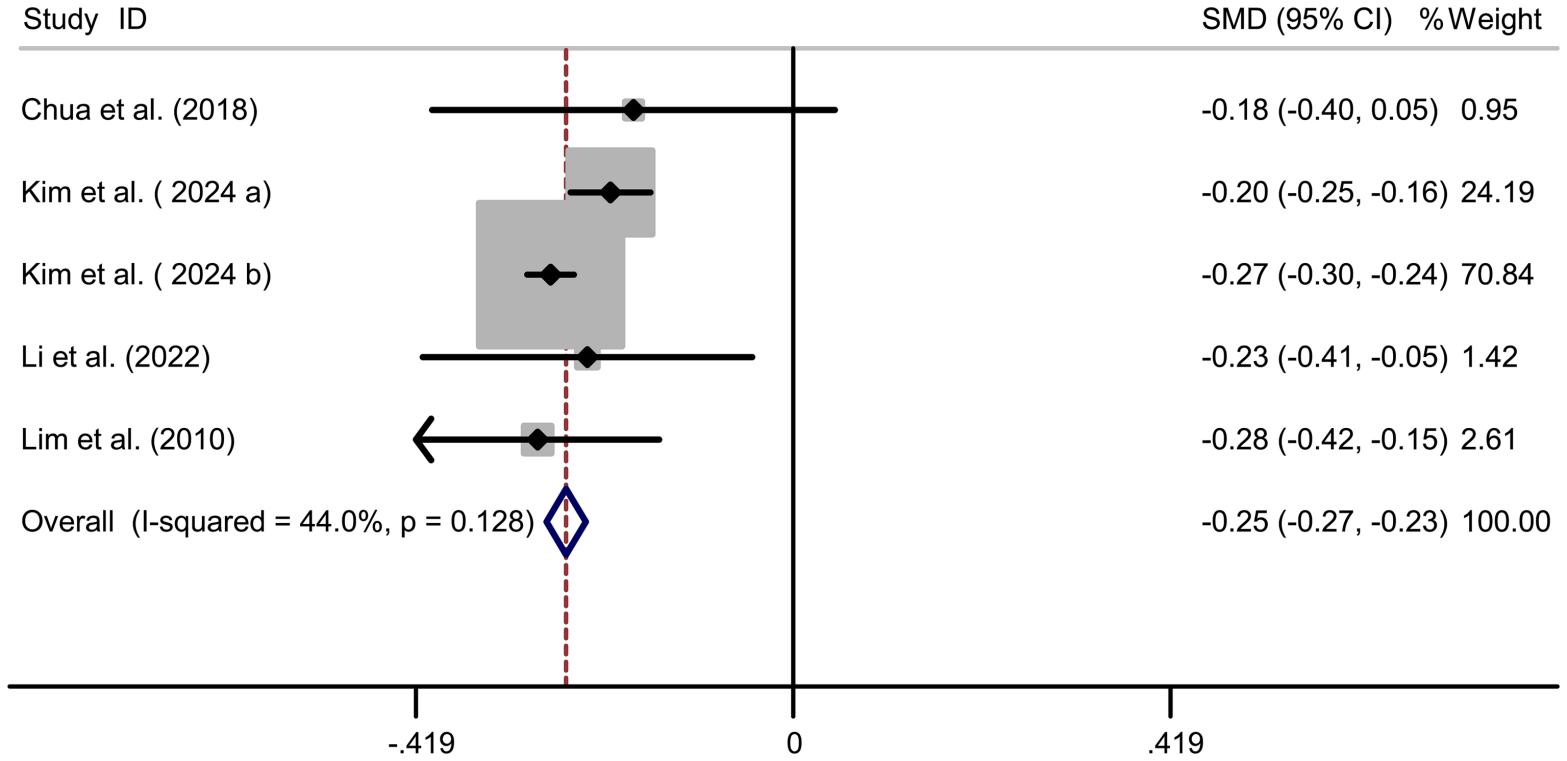
Forest plot of protein intake and myopia risk.

**Figure 5:**
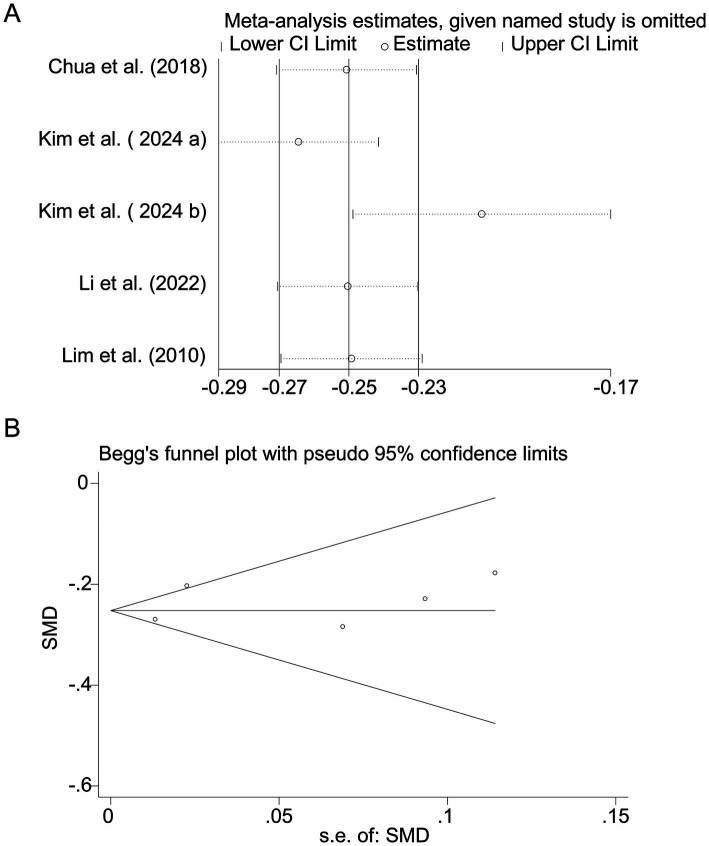
Sensitivity analysis and funnel plot for protein intake. **(A)** Sensitivity analysis for protein intake. **(B)** Funnel plot for protein intake publication bias.

Pooled analysis of 5 studies (17, 18, 20–22) demonstrated a protective effect of protein intake against myopia (SMD = −0.25, 95% CI: −0.27−−0.23, I^2^ = 44.0%, *p* < 0.001; Figure 4). Although heterogeneity was moderate (I^2^ = 44.0%), subgroup analyses showed that the protective association of protein intake remained stable across different study designs and regions. Animal-based protein–dominant studies demonstrated slightly stronger protective effects (SMD = −0.28, 95% CI: −0.33 to −0.23; I^2^ = 39.2%) compared with mixed or plant-based protein studies (SMD = −0.22, 95% CI: −0.27 to −0.18; I^2^ = 41.6%). Sensitivity analysis confirmed consistency across studies (Figure 5A), and no publication bias was detected (Begg’s test, *p* = 0.87; Figure 5B).

### Cholesterol intake and myopia risk

**Figure 6:**
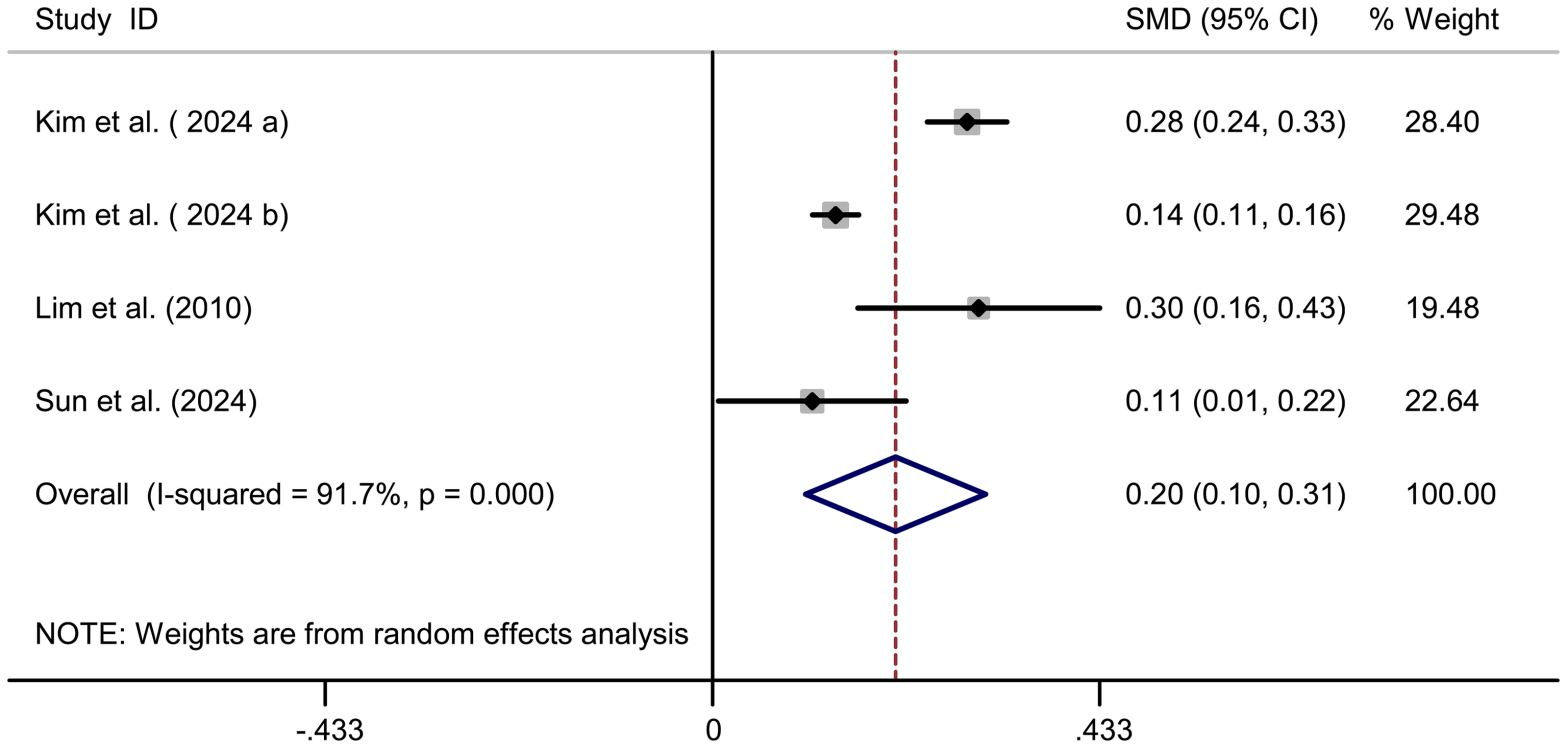
Forest plot of cholesterol intake and myopia risk.

**Figure 7:**
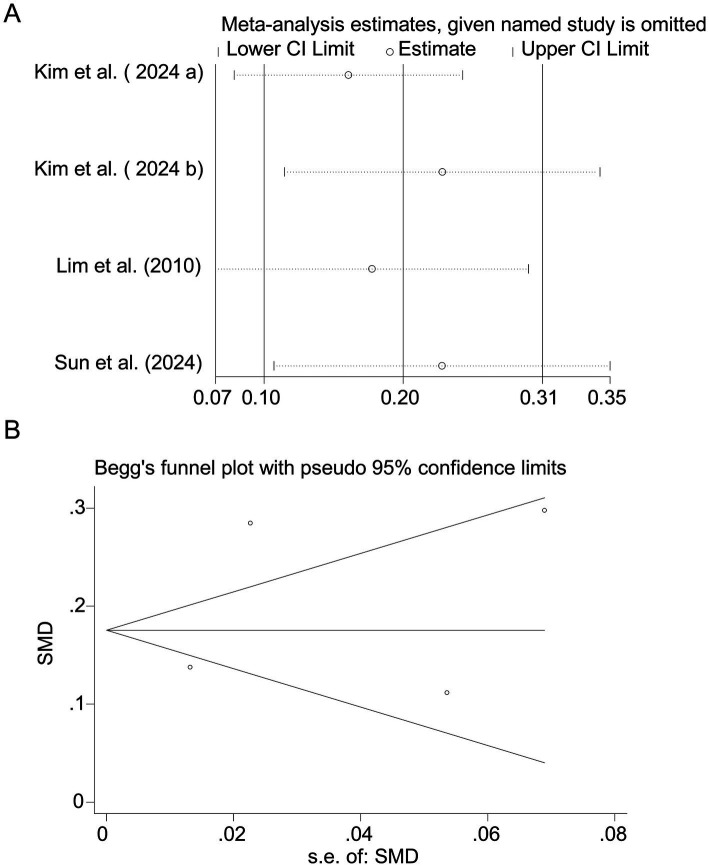
Sensitivity analysis and funnel plot for cholesterol intake. **(A)** Sensitivity analysis for cholesterol intake. **(B)** Funnel plot for cholesterol intake publication bias.

Four studies (17–19, 22) showed a significant positive association between cholesterol intake and myopia risk (SMD = 0.20, 95% CI: 0.10–0.31, I^2^ = 91.7%, *p* < 0.001; Figure 6). Sensitivity analysis supported the robustness of the effect (Figure 7A), and no publication bias was evident (Begg’s test, *p* = 0.53; Figure 7B). The high heterogeneity (I^2^ = 91.7%) was substantially influenced by dietary assessment methods. In studies using 24-h dietary recalls, the association between cholesterol intake and myopia was more consistent (SMD = 0.22, 95% CI: 0.15–0.30; I^2^ = 72.4%), whereas questionnaire-based studies exhibited extreme variability (SMD = 0.19, 95% CI: −0.05–0.43; I^2^ = 93.5%). Regional differences also contributed, with East Asian populations showing stronger associations than Western cohorts.

### Sodium intake and myopia risk

**Figure 8:**
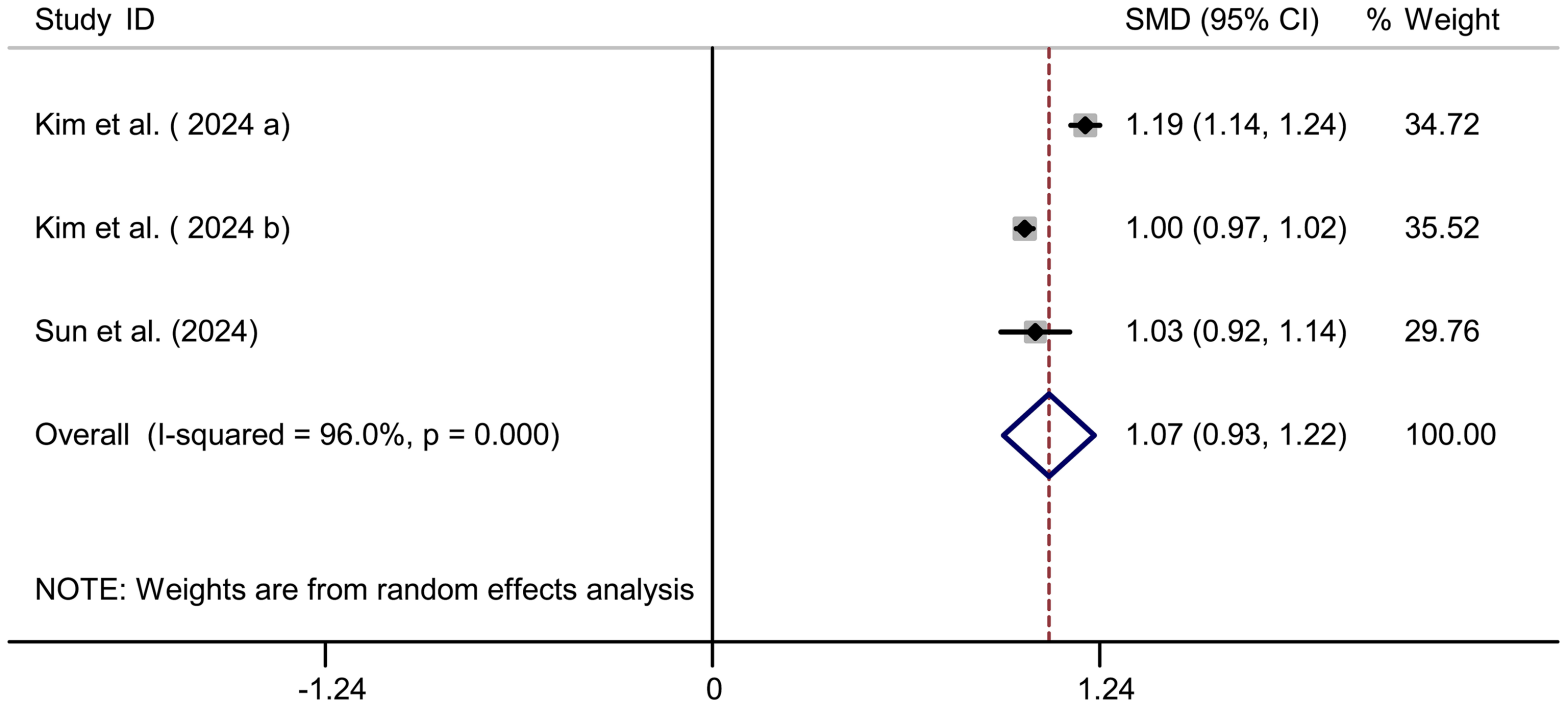
Forest plot of sodium intake and myopia risk.

**Figure 9:**
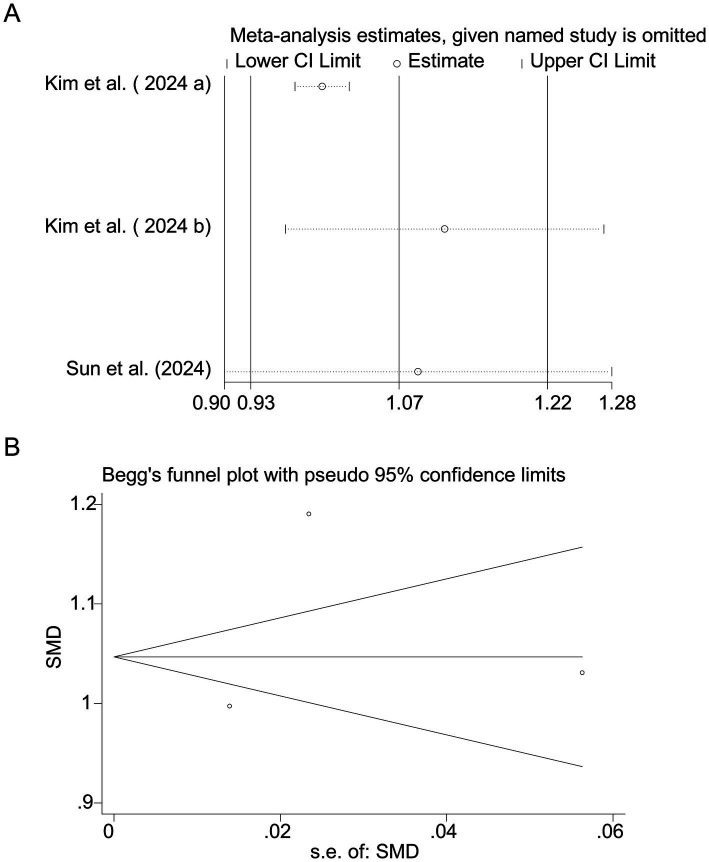
Sensitivity analysis and funnel plot for sodium intake. **(A)** Sensitivity analysis for sodium intake. **(B)** Funnel plot for sodium intake publication bias.

Pooled results from 3 studies (17–19) indicated a strong positive association between sodium intake and myopia (SMD = 1.07, 95% CI: 0.93–1.22, I^2^ = 96.0%, *p* < 0.001; Figure 8). Extreme heterogeneity likely stemmed from regional dietary habits (e.g., Korean *vs.* Chinese populations). Sensitivity analysis confirmed stable results (Figure 9A), and publication bias was absent (Begg’s test, *p* = 0.65; Figure 9B).

## Discussion

Adolescent myopia has reached epidemic levels, particularly in East Asia, where prevalence rates exceed 30–50% (4, 11, 13). This rising trend is concerning due to the increased risk of sight-threatening complications such as retinal detachment and glaucoma (23). While genetic predisposition and environmental factors—including prolonged near work and reduced outdoor activity—are well-established contributors, our findings highlight the role of nutrition as a modifiable risk factor. Dietary modifications during adolescence could therefore serve as a complementary strategy to existing myopia control interventions, such as orthokeratology and atropine therapy.

Our meta-analysis identified dietary carbohydrates, cholesterol, and sodium as potential risk factors for myopia, whereas protein intake appeared to confer a protective effect. These associations align with proposed biological mechanisms. For instance, excessive carbohydrate intake may elevate insulin-like growth factor 1 (IGF-1) levels, thereby promoting scleral remodeling and axial elongation (24). Another hypothesized pathway is that high sodium intake may cause osmotic stress and disturb intraocular fluid balance, potentially contributing to axial elongation. Nutritional epidemiology provides some supportive human evidence: high dietary sodium has been linked to approximately a twofold increase in myopia risk in adolescents, and populations with traditionally low-salt diets (e.g., Amazonian natives) exhibit lower myopia prevalence (23). However, the causal role of sodium remains speculative, as longitudinal or interventional studies in youth are scarce. Conversely, proteins rich in essential amino acids (e.g., lysine, proline) may support collagen synthesis, enhancing scleral rigidity and resisting axial elongation (7). Importantly, subgroup analyses provided additional insights into the substantial heterogeneity observed in the main analyses. For carbohydrate intake, regional differences and dietary assessment methods explained a large proportion of variability: East Asian studies, characterized by higher refined carbohydrate consumption, demonstrated stronger associations than Western cohorts, while studies employing semi-quantitative diaries or 24-h recalls showed more consistent findings than those using food frequency questionnaires. For protein intake, the protective effect was stable across subgroups, but slightly stronger in studies emphasizing animal-based sources. Similarly, the heterogeneity in cholesterol and sodium analyses was substantially reduced when stratified by dietary assessment method, with 24-h recalls yielding more homogeneous results compared with questionnaire-based assessments. Regional dietary patterns, particularly the traditionally high sodium intake in Korea and China, also contributed to the heterogeneity. These subgroup findings strengthen the robustness of our results and highlight the influence of methodological and cultural factors on observed associations.

Quantitative synthesis indicated that higher carbohydrate intake was associated with increased myopia risk (SMD = 0.36, 95% CI: 0.22–0.50), particularly in East Asian cohorts. Subgroup analyses suggested that both study region and dietary assessment methods contributed to the observed heterogeneity. One possible mechanism is that excessive carbohydrate intake may influence insulin-like growth factor-1 (IGF-1) signaling, thereby promoting scleral remodeling and axial elongation. Some human data provide indirect support: IGF-1 gene polymorphisms have been associated with myopia susceptibility, and IGF-1/STAT3 pathways have been implicated in scleral remodeling in experimental studies (25, 26). Nevertheless, these mechanisms remain provisional, as direct confirmation in adolescent cohorts is limited.

Similarly, our pooled analysis demonstrated a protective association of protein intake with myopia (SMD = −0.25, 95% CI: −0.27 to −0.23), with relatively low heterogeneity (I^2^ = 44.0%). This protective effect was observed consistently across study designs and regions. A possible explanation is that protein intake, particularly rich in amino acids like lysine and proline, may support collagen synthesis and scleral rigidity, thereby reducing susceptibility to axial elongation. While this rationale is biologically plausible, current evidence is mostly experimental or animal-based, with limited direct validation in adolescent populations (27, 28). Beyond supporting collagen synthesis and scleral rigidity, dietary proteins could exert broader protective effects against myopia through their influence on skeletal growth, body composition, and energy metabolism. Adequate protein intake is essential for normal skeletal development during adolescence, a period of rapid axial elongation; balanced skeletal growth may help maintain proportional ocular development and reduce the tendency toward excessive axial lengthening (29). Proteins also modulate body composition by promoting lean muscle mass over adiposity, which is relevant because higher adiposity has been linked to metabolic disturbances and myopia risk (30). In addition, proteins contribute to efficient energy metabolism and glycemic regulation, potentially counteracting hyperinsulinemia and IGF-1 dysregulation that drive scleral remodeling (31). Together, these pathways suggest that sufficient protein intake may create a systemic environment less conducive to pathological eye growth, thereby offering a plausible biological basis for its protective association with myopia. Nevertheless, these pathways are hypothetical and require validation in experimental or longitudinal studies.

In addition to the four nutritional factors evaluated in our meta-analysis, other dietary components have also been investigated in relation to myopia (5, 32). For example, several large cross-sectional studies and meta-analyses have examined vitamin D, while others have focused on antioxidants such as anthocyanins and polyunsaturated fatty acids (5, 32). These nutrients may exert protective effects through anti-inflammatory and antioxidative mechanisms, as well as maintenance of scleral extracellular matrix integrity. However, despite the relatively larger body of work on vitamin D, the evidence across these nutrients remains inconsistent, with heterogeneity in study design, exposure measurement, and confounder adjustment. Our study therefore focused on carbohydrates, proteins, cholesterol, and sodium, which had sufficient comparable data for quantitative synthesis, but future work should incorporate these additional nutrients to build a more comprehensive understanding of the dietary determinants of myopia.

The associations between myopia and both cholesterol (SMD = 0.20, 95% CI: 0.10–0.31) and sodium (SMD = 1.07, 95% CI: 0.93–1.22) warrant cautious interpretation. High cholesterol intake has been speculated to promote ocular inflammation and oxidative stress, thereby contributing to myopia development. However, evidence in adolescents remains indirect, primarily from cross-sectional associations, and mechanistic data at the human level are scarce. These findings should therefore be interpreted cautiously until confirmed in prospective studies. However, the substantial heterogeneity in sodium-related studies (I^2^ = 96.0%) highlights the need for culturally specific dietary guidelines, as sodium intake varies significantly across populations (e.g., Korean diets typically contain higher sodium levels than Western diets). Although subgroup analyses helped to identify potential sources of heterogeneity—such as study design, dietary assessment method, and regional dietary patterns—residual heterogeneity remained high in some comparisons. This suggests that additional unmeasured factors, such as differences in carbohydrate quality, protein sources, cooking practices, or unadjusted confounders (e.g., parental myopia, physical activity), may further contribute to the variability. Future studies should incorporate standardized dietary assessment tools and harmonized outcome definitions to minimize these sources of heterogeneity.

This meta-analysis systematically synthesized evidence from eight studies, providing robust effect estimates for key nutrients. Our rigorous inclusion criteria, quality assessment (NOS scores ≥6), and sensitivity analyses strengthened the reliability of our conclusions. However, substantial heterogeneity across studies—arising from differences in dietary assessment methods, myopia definitions, and study designs—may limit the generalizability of our findings. Most included studies were cross-sectional, precluding causal inferences. Another important limitation is that key confounders such as obesity, socioeconomic status, parental myopia, and physical activity were not consistently adjusted for across the included studies. These factors are well-established contributors to myopia risk and may interact with dietary intake, potentially leading to residual confounding in our pooled estimates. For instance, obesity is associated with both dietary patterns and refractive development, while parental myopia strongly influences genetic susceptibility. Therefore, the observed associations between nutrition and myopia should be interpreted with caution. In addition, residual confounding by other unmeasured factors (e.g., differences in carbohydrate quality, protein sources, cooking practices) and variations in nutrient bioavailability may also have influenced the results. To establish causality, future research should prioritize longitudinal studies with validated dietary assessments and comprehensive adjustment for these confounders, as well as investigations into gene-diet interactions and clinical trials testing dietary interventions.

To establish causality, future research should prioritize longitudinal studies with validated dietary assessments, such as 24-h recalls combined with biomarkers. Additionally, investigations into gene-diet interactions (e.g., polymorphisms in IGF-1 signaling pathways) could provide insights into individual susceptibility to diet-induced myopia progression. Clinical trials evaluating dietary interventions, such as low-carbohydrate, high-protein diets, may further clarify the role of nutrition in myopia management. Nevertheless, the evidence base is limited, with only eight observational studies included—most of which are cross-sectional—severely restricting causal inference. Therefore, our results should not be interpreted as direct evidence to support policy-level dietary recommendations. At this stage, nutritional factors can be viewed as potential modifiable correlates rather than established causal determinants of myopia. Future large-scale, prospective cohort studies and randomized controlled trials are warranted to provide higher-level evidence.

## Data Availability

The data analyzed in this study is subject to the following licenses/restrictions: the data that support the findings of this study are available from the corresponding author upon reasonable request. Requests to access these datasets should be directed to ZX, 274278961@hbust.edu.cn.
